# Low-Protein Diets Could Be Effective and Safe in Elderly Patients with Advanced Diabetic Kidney Disease

**DOI:** 10.3390/nu16142230

**Published:** 2024-07-11

**Authors:** Liliana Garneata, Carmen-Antonia Mocanu, Gabriel Mircescu

**Affiliations:** 1 Department of Internal Medicine and Nephrology, “Carol Davila” University of Medicine and Pharmacy, 050474 Bucharest, Romania; 2 Department of Nephrology, “Dr. Carol Davila” Teaching Hospital of Nephrology, 010731 Bucharest, Romania

**Keywords:** chronic kidney disease, diabetic kidney disease, elderly, low-protein diet

## Abstract

Low-protein diets (LPDs) seem to improve metabolic complications of advanced CKD, thus postponing kidney replacement therapy (KRT) initiation. However, the nutritional safety of LPDs remains debatable in patients with diabetic kidney disease (DKD), especially in the elderly. This is a sub-analysis of a prospective unicentric interventional study which assessed the effects of LPD in patients with advanced DKD, focusing on the feasibility and safety of LPD in elderly patients. Ninety-two patients with DKD and stable CKD stage 4+, proteinuria >3 g/g creatininuria, good nutritional status, with confirmed compliance to protein restriction, were enrolled and received LPD (0.6 g mixed proteins/kg-day) supplemented with ketoanalogues of essential amino acids for 12 months. Of the total group, 42% were elderly with a median eGFR 12.6 mL/min and a median proteinuria 5.14 g/g creatininuria. In elderly patients, proteinuria decreased by 70% compared to baseline. The rate of kidney function decline was 0.1 versus 0.5 mL/min-month before enrolment. Vascular events occurred in 15% of cases, not related to nutritional intervention, but to the severity of CKD and higher MAP. LPDs seem to be safe and effective in postponing KRT in elderly patients with advanced DKD while preserving the nutritional status.

## 1. Introduction

Type 2 diabetes mellitus (T2DM) has an increasing prevalence all over the world, and is associated in 40–50% of cases with chronic kidney disease (CKD) [1,2]. The association of diabetes with CKD is defined as diabetic kidney disease (DKD) and in its latter stages is characterized by a particularly rapid decline in kidney function, frequently leading to kidney replacement therapy (KRT) [3,4]. Moreover, DKD is a major risk factor of cardiovascular disease (CVD).

The prevalence of DKD increases with age. It is estimated at 4.4% in patients <40 years old, 32% between 65 and 75 years, and 61% over the age of 75 [5]. The progression of decline in eGFR, the increase in proteinuria and the risk of KRT are independently related to age in patients over 75 years old [6].

Although improved metabolic and blood pressure control allowed for a reduction in KRT of 3% per year in DKD patients [7], nearly all pivotal studies evaluating the efficacy of new therapies were performed in cohorts of relatively young patients. Moreover, patients >70 years were excluded. Accordingly, the management of elderly DKD patients is based on extrapolations of data gathered from younger patients [8].

Thus, beside the specific therapy for blood glucose control, there is a significant interest in preventing kidney injury, as well as postponing KRT using a multifactorial approach: control of cardiovascular risk factors (i.e., blood pressure, dyslipidemia), along with lifestyle changes (diet, exercise, and smoking cessation) [9,10].

Nutritional intervention has been implemented in patients with CKD and results seem favorable in reducing kidney function decline [11], as well as proteinuria [12]. Another challenge associated with DKD is the safety of LPDs in patients who already require a reduced carbohydrate intake [13].

In non-diabetic patients with CKD, very low-protein diets (VLPDs) supplemented with ketoanalogues of essential amino acids (KA) seem to improve metabolic abnormalities associated with advanced kidney disease, i.e., nitrogenous waste retention, acid-base balance disorders, and mineral and bone disorders [12,14,15,16,17]. Other studies suggest benefits of a vegetarian VLPD supplemented with KA by lowering insulin resistance, improving lipid metabolism and lowering albuminuria as well as the rate of kidney function decline [18,19]. There are few studies that assess the benefits and risks of LPD in elderly patients [20].

On the other hand, patients with DKD, especially the elderly, have a high prevalence of cardiovascular and cerebrovascular disease [21]. Likewise, older patients have a higher risk of developing orthostatic hypotension. Therefore, aggressive blood pressure control may increase cerebrovascular morbidity and mortality as well as incidence of acute kidney injury [22].

Moreover, older patients have a higher risk of malnutrition. Thus, implementing an LPD may be challenging and increase the risk of muscle-mass loss [23].

Therefore, based on the positive results of LPD for younger patients with high-risk chronic kidney disease, it is also important to know the direct effects of low-protein diets in elderly patients with advanced diabetic kidney disease. This study aims to investigate the effectiveness and applicability of nutritional intervention in CKD in this group of patients.

## 2. Materials and Methods

### 2.1. Design

**Figure 1:**
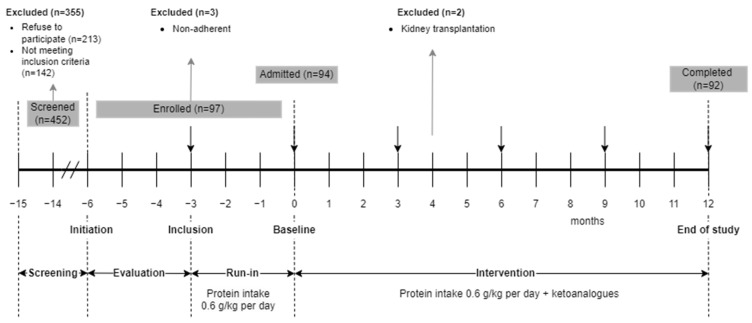
Study design.

This is a sub-analysis of an interventional prospective unicentric study with a 15-month follow-up duration divided into four phases (Figure 1).

In the screening phase, all patients with T2DM and CKD from a tertiary nephrology single-center admitted in one year were evaluated for eligibility. Patients who met the selection criteria and agreed to the nutritional intervention were enrolled into the Run-In phase. Patients also received the standard of care treatment for DKD, according to current guidelines [24].

In the Run-In phase, patients received intensive nutritional counselling regarding a protein-restricted, mostly vegetarian LPD. If patients remained adherent to the prescribed dietary regimen, they entered the intervention phase.

The intervention phase had a 12-month duration and consisted of maintaining an LPD supplemented with KA. Compliance to the intervention, efficacy, secondary, and safety parameters were monitored monthly.

### 2.2. Ethics

The study was conducted in accordance with the Declaration of Helsinki, approved by the local Ethics Committee of “Dr. Carol Davila” Teaching Hospital of Nephrology, Bucharest, Romania (Committee’s Reference number 124/2013) and registered in the National Clinical Trials database (NCT 03415074).

Data regarding the effects of the LPD diet on eGFR, proteinuria, and blood pressure have been published previously [25,26].

### 2.3. Selection Criteria

Adult patients (>18 years old) with T2DM and high proteinuria were considered for enrollment. The inclusion criteria were adult age, T2DM, stage 4+ CKD [24] (estimated glomerular filtration rate—eGFR < 30 mL/min/1.73 m^2^, MDRD4 equation [27]), urinary protein to creatinine ratio >3 g/g, a good nutritional status (Subjective Global Assessment Score—SGA A [28], serum albumin > 3.5 g/dL) with less than 5% variation during the evaluation phase. Before enrollment, patients were informed regarding the prerequisite of following a mostly vegetarian LPD.

Patients with active kidney disease, necessitating specific therapy (rapid increase in proteinuria, acute decline in eGFR, dysmorphic hematuria), those without diabetic microangiopathy, and patients with severe comorbidities (heart failure, severe peripheral artery disease, liver cirrhosis, malabsorption, on-going infections, inflammatory disease requiring corticosteroids or immunosuppressive therapy and uremia—pericarditis, gastrointestinal disorders, bleeding) were excluded from the study.

### 2.4. Intervention

The nutritional intervention was a conventional mixed LPD (0.6 g/kg-day), mainly vegetarian, supplemented with KA (Ketosteril^®^ Fresenius Kabi, Bad Homburg, Germany) 1/tb/10 kg-dry body weight, divided in three doses per day, taken with meals. The choice of vegetables, fruits, legumes, and cereals was left at the patients’ discretion. To improve adherence, 5 meals that included animal-based protein were allowed per week.

A carbohydrates intake of 200 g/day was recommended; antidiabetic treatment was adjusted by the diabetologist.

The recommended daily calorie intake was 25–30 kcal/kg (dry-body weight), individualized according to patient age, gender, current BMI, level of physical activity.

Conventional management of CKD was conducted in accordance to national guidelines [10,24].

High blood pressure and hypercholesterolemia were treated with antihypertensive medication (including renin–angiotensin–aldosterone inhibitors, RAASI) and statins, respectively. Iron supplements and erythropoietin stimulating agents were administered as needed [29]. Mineral and bone disorders were corrected with calcium supplements, phosphate binders, and vitamin D administration [30].

Patients received intensive nutritional counselling and were monitored monthly during the Run-In phase. Additionally, on starting the intervention phase, the regimen was further intensified to once every other week during the first month, followed by monthly for the next 3 months and every three months thereafter.

### 2.5. Parameters

The primary efficacy endpoint of the study was the dynamic of proteinuria during the intervention.

In this sub-analysis, we focused on the efficacy, feasibility, and last but not least, safety in elderly patients (>65 years old).

Efficacy parameters were considered as follows: the reduction in proteinuria and the variation of kidney function from baseline to end of study (EOS) in elderly patients. Proteinuria was measured from a 24 h collection sample and expressed as g/g of urinary creatinine. Kidney function was expressed as eGFR calculated using the MDRD4 formula [27].

Secondary parameters were the variation of blood pressure and the occurrence of vascular events throughout the study. Systolic and diastolic blood pressure (SBP, DBP) were measured at each visit, according to the ESH/ESC guidelines [31] and mean arterial blood pressure (MAP) was computed as MAP = DBP + 1/3(SBP − DBP). Hypertension was defined as either BP over 140/90 mmHg or use of antihypertensive medication. The attending physician was free to adjust the antihypertensive medication: RAASi, calcium channel- and beta-blockers, as well as loop diuretics (furosemide), targeting a 130/80 mmHg BP [31,32]. Vascular events included major cardiovascular events (acute coronary syndrome, coronary revascularization, congestive heart failure, or peripheral vascular events) or cerebrovascular events (ischemic or hemorrhagic stroke or transient cerebral ischemia).

Safety parameters were focused on the nutritional status (SGA), body mass index (BMI), serum albumin, as well as inflammation (C-reactive protein—CRP) and glucose metabolism control (glycated hemoglobin—HbA1c%).

Patient diet adherence was assessed through the estimated protein intake by urinary excretion of urea (Mitch-Maroni formula [33]). The energy intake was assessed by a 3-day food diary. Patients with variations greater than ±10% in either protein, carbohydrates, or energy intake were considered non-compliant and therefore excluded from the study.

Data obtained at inclusion, baseline, 3, 6, 9 months, and at EOS were included in the analysis.

### 2.6. Statistical Analysis

Data from elderly patients were compared to data from non-elderly patients.

Slopes of proteinuria and eGFR in study phases were calculated using linear regression.

Continuous parameters are presented as median and 95% confidence intervals (95% CI) of the median. The type of distribution was assessed by the Shapiro–Wilk test. Categorical parameters were expressed as percentages.

Comparisons for grouped data were evaluated by the Wilcoxon–Mann–Whitney test for non-parametric data and by grouped *T*-test for those with a normal distribution. For categorical data, the comparisons were made using the Pearson Chi-square test. Comparisons for paired data were evaluated by the Friedman test for non-parametric data and paired *T*-test for parametric data. For categorical data comparisons were made using the McNemar test. Differences between paired data were evaluated using the Sign test.

This sub-analysis has a repeated measurement design.

Univariate correlations were performed using Kendall’s tau-test.

The interaction of eGFR and proteinuria by study moments and group age were described using a two-way ANOVA model after the log transformation of the dependent variables.

The determinants associated with proteinuria and with eGFR were analyzed in models of multiple linear regression (elderly versus non-elderly) after log transformation of all the included variables.

Binary regression was used to assess the determinants of vascular events at inclusion, baseline, month 3, month 6, month 9, and EOS after optimizing the accuracy using score Z for all the included variables.

A *p*-value below 0.05 was considered statistically significant.

Statistical analysis was performed using Analyse-it version 6 (Analyse-it Software, Ltd., Leeds, UK) and IBM SPSS version 25 (IBM, New York, NY, USA).

## 3. Results

Of the 452 screened patients, 213 refused to participate and 142 did not meet the selection criteria. Only 97 patients (21% of 452) were enrolled in the Run-In phase. Three patients were not adherent to the LPD and were excluded. During the study, 2 patients received a preemptive kidney transplant. The possibility of the kidney transplant was constrained by graft availability, not patient necessity.

The data collected from the 92 patients completing all study phases was used in obtaining the final results (Figure 1).

Thirty-nine (42% of 92) were elderly and all of them completed the study. None of the included subjects required KRT during the study. Median age of elderly people was 75 years old and 64% of them were men (Table 1).

**Figure 2:**
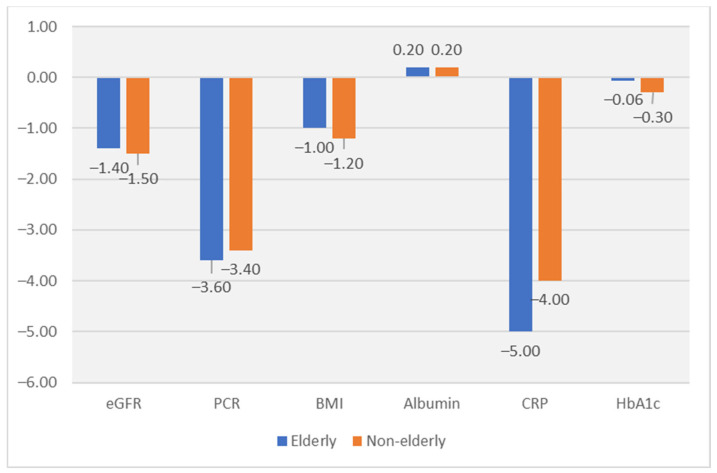
Difference (EOS–Baseline) in the Intervention phase. All differences EOS–Baseline are significant for all parameters, but there are no significant differences between age groups for any parameter. Albumin (serum albumin; g/dL); BMI (Body mass index; kg/m^2^); CRP (C-reactive protein; mg/L); eGFR (estimated glomerular filtration rate; mL/min); Hb1c (glucated hemoglobin; %); Proteinuria (PCR; g/g).

When comparing elderly with non-elderly patients, overall, there were no significant differences at study moments in terms of the studied variables (Table 2 and Table 3, Figure 2).

### 3.1. Efficacy Parameters in Elderly Patients

#### 3.1.1. Proteinuria

At inclusion, the median proteinuria in elderly patients was 4.8 g/g urinary creatinine (Table 1). In the intervention phase, proteinuria significantly decreased in both groups but without differences between groups (−3.6 g/g in elderly; −3.4 g/g in non-elderly) (Table 3, Figure 2).

Slopes of protein-to-creatinine ratio (PCR) in the Run-In (Inclusion–Baseline) and the Intervention (Baseline–EOS) were calculated. The PCR decreased significantly, without differences between groups, from 0.00 (−0.40 to 0.17) to −0.32 (−0.38 to −0.27) g/g per month in the elderly subgroup, and from −0.05 (−0.37 to 0.17) to −0.29 (−0.32 to −0.26) g/g per month in non-elderly (Table 2 and Table 3, Figure 2).

A two-way ANOVA was conducted that examined the effect of age group (elderly versus non-elderly) and study moment (Run-In versus Intervention) on proteinuria. Although the intervention was significantly correlated with the dependent variable (F(1,548) = 106.57, *p* < 0.001), age did not have any effect on the model (F(1,548) = 0.58, *p* = 0.45). There was no statistically significant interaction between the effects of study moment and group age on proteinuria (Table 4).

Using correlation analysis, PCR was directly related to eGFR, estimated protein intake, mean arterial pressure, glycated hemoglobin, and BMI. We further tested the effects of age on factors associated with proteinuria in a multivariate linear regression. In both age groups, eGFR (R^2^ = 0.48, Beta = 0.86, *p* < 0.0001 in elderly and R^2^ = 0.49, Beta = 0.71, *p* < 0.0001 in non-elderly) and higher protein intake (R^2^ = 0.48, Beta = 0.75, *p* < 0.0001 in elderly and R^2^ = 0.49, Beta = 0.57, *p* = 0.001 in non-elderly) were independently associated to higher proteinuria. Higher glycated hemoglobin (only in elderly: R^2^ = 0.48, Beta = 0.64, *p* = 0.04) and higher BMI (only in non-elderly: R^2^ = 0.49, Beta = 1.28, *p* = 0.001) were independently related to a higher proteinuria (Table 5).

#### 3.1.2. Estimated Glomerular Filtration Rate

At inclusion, median eGFR was 11.7 mL/min with a slight increase by (0.9 mL/min) at baseline, probably in accordance to the introduction of the LPD (Table 1 and Table 2). During the 12 months intervention, the eGFR decreased by 1.5 mL/min (Table 3), with no differences between groups (Table 2 and Table 3, Figure 2).

Slopes of eGFR in the Evaluation phase (Initiation–Inclusion), Run-In phase (Inclusion–Baseline) and the Intervention phase (Baseline–EOS) were calculated. During the intervention, the decline of eGFR was reduced by 80% (Table 1 and Table 2). The rate of eGFR decline was halved without differences between groups, from 0.22 (0.16 to 0.41) to −0.12 (−0.25 to −0.08) mL/min per month in the elderly, and from 0.20 (0.14 to 0.49) to −0.10 (−0.15 to −0.08) mL/min per year in non-elderly (Table 2).

A two-way ANOVA was conducted that examined the effect of age group (elderly versus non-elderly) and study moment (Run-In versus Intervention) on eGFR. Although the intervention was significantly correlated with the dependent variable (F(1,548) = 5.39, *p* = 0.02), age did not have any effect on the model (F(1,548) = 1.92, *p* = 0.17). There was no statistically significant interaction between the effects of study moment and group age on eGFR (Table 4).

Using correlation analysis, eGFR was related to age, MAP, proteinuria, and protein intake. We further examined the effects of age on factors associated to eGFR in a multivariate linear regression (elderly vs. non-elderly). eGFR was independently and directly associated with proteinuria (R^2^ = 0.41, Beta = 0.47, *p* < 0.0001 in elderly and R^2^ = 0.36, Beta = 0.60, *p* < 0.0001 in non-elderly), but only in the elderly subgroup there was an inverse association to protein intake (R^2^ = 0.41, Beta = −0.34, *p* = 0.02) (Table 6). Hence, LPD seems to limit reduction in eGFR decline but only in elderly patients.

### 3.2. Secondary Parameters in Elderly Patients

#### 3.2.1. Blood Pressure

At inclusion, median SBP was 130 mmHg and median DBP was 70 mmHg with a MAP of 93 mmHg (Table 1).

SBP and MAP were similar between elderly and non-elderly patients (Table 2). Although at baseline there were differences between subgroups in DBP, those differences were attenuated during the study period (Table 2). At EOS, SBP decreased by 10 mmHg, and DBP by 10 mmHg. The median blood pressure at EOS was 130/60 mmHg, and MAP decreased by 11 mmHg (Table 3).

The antihypertensive therapy changed in the Intervention phase, similarly in both age groups. The proportion of patients treated with RAASi decreased (−31% and −21%), and the proportion of those treated with furosemide increased (31% and 21%) in elderly and non-elderly, respectively, without effect on blood pressure control, suggesting appropriate adjustment of antihypertensive therapy (Table 3).

None of the patients were treated with Sodium-Glucose Transport Protein 2 Inhibitors.

#### 3.2.2. Vascular Events

At inclusion and at baseline, none of the patients had vascular events. However, during the study, a few patients experienced cardiovascular or cerebrovascular events. Non-elderly patients seemed to have a higher risk of cardiovascular events, but the results are influenced by the small number of the events (Table 2). However, there were no significant differences between elderly and non-elderly patients regarding the risk of all vascular events (Table 2).

During the study, 15% of the elderly patients experienced vascular events (Table 3). Assessing the potential determinants of the vascular events, only MAP was directly associated with a higher risk, while eGFR was inversely associated with the risk (Table 7).

### 3.3. Safety Parameters in Elderly Patients

All patients had SGA score A at all study moments (Table 2 and Table 3).

In elderly patients, nutritional and inflammatory status improved. Serum albumin increased by 0.3 g/dL, from 3.9 g/dL to 4.2 (Table 2 and Table 3, Figure 2). CRP decreased by 5 mg/L during the study period (Table 3, Figure 2). BMI decreased from 27.1 kg/m^2^ to 25.7 (Table 2, Figure 2). The management of carbohydrate metabolism was not influenced by dietary interventions. HbA1c levels did not change during the study (Table 2 and Table 3, Figure 2).

The achieved energy intake was close to the prescription (31 kcal/kg per day), without changes during the study (Table 3) and without difference between elderly and non-elderly patients.

In terms of the safety parameters, there were no differences at any of the study moments between elderly and non-elderly patients (Table 2).

### 3.4. Adherence to the Intervention

**Figure 3:**
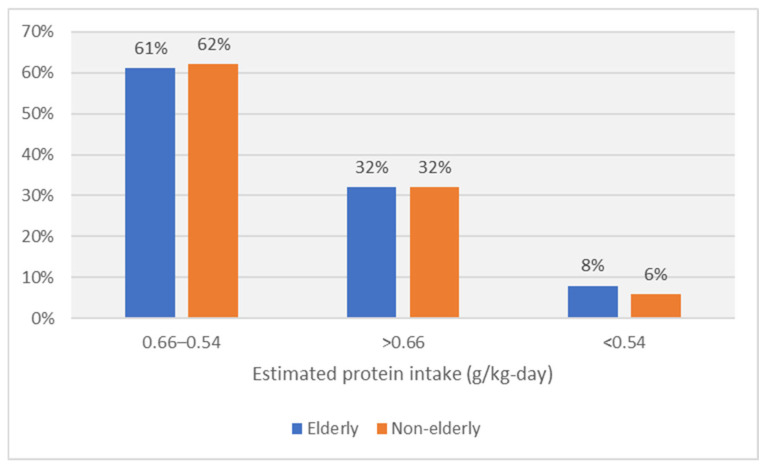
Adherence to recommended protein intake in the Intervention phase by age group.

At inclusion, the median estimated protein intake was 0.9 g/kg per day and decreased, as per recommendation, to 0.69 g/kg per day at baseline (Table 1 and Table 2). During the study, compliance to LPD significantly increased, with a difference in the estimated protein intake of −0.03 g/kg per day between EOS and baseline (Figure 2). In the Intervention phase, in 61% of cases the estimated protein intake was between 0.54 and 0.66 g/kg-day in elderly, and only in 8% cases the estimated protein intake (EPI) was lower than 0.54 g/kg-day (Figure 3).

The average estimated protein intake was similar to the one prescribed throughout the study. The compliance to the LPD increased by 39% during the study (Table 3).

There were no differences between elderly and non-elderly patients regarding diet adherence and compliance to the intervention (Table 3, Figure 2).

## 4. Discussion

We have assessed the efficacy, feasibility, and safety of LPD supplemented with KA in elderly patients with advanced DKD. This study is among the few published that not only investigate the effects of LPD in advanced DKD, but also its effects on elderly patients.

Data on the efficacy and safety of LPDs in patients with DKD are conflicting. This is particularly important in elderly patients due to the increased risk of malnutrition [34,35,36,37,38]. Currently, the average estimated requirement for dietary protein is 0.66 g/kg per day with a recommended dietary allowance of 0.8 g/kg per day for all adults over 18 years old, including elderly adults [39].

The major challenge of the LPD is in patients with DM, already on carbohydrate restriction, especially in elderly, who are less likely to change their dietary habits and, occasionally, have less family support [40,41]. To improve adherence, as well as to maintain the nutritional requirements for elderly patients with DKD, five meals with animal-based protein origin can be allowed per week [42]. In this study, it is important to mention that only 21% of the screened patients were compliant to LPD and entered the study. Although the number of participants was small, the patients who entered the study remained adherent to the LPD, with an improvement of protein restriction adherence by 39% during the study. This result was related to nutritional and clinical counselling.

We are reporting an important reduction in proteinuria by 3.6 g/g creatininuria and a remarkable 5-fold reduction in the decline of eGFR with a difference of 1.5 mL/min in 15 months of intervention, similar to the physiological decline of eGFR [43]. In this study, these findings were similarly noticed in non-elderly patients.

As this reduction in proteinuria was found in patients treated with RAASi for the most part of the Intervention phase, LPD seem to have an additive effect to RAASi on proteinuria, as previously noted [44].

Proteinuria was directly related to EPI and to eGFR, BMI, and glycemic control (HbA1c). However, the effects of LPD on proteinuria seem to differ by age group. In the elderly group, HbA1c was not independently related to proteinuria, suggesting that HbA1c control was better in this age group, while in the non-elderly groups, proteinuria was independently related to BMI.

The estimated glomerular filtration rate similarly decreased with only 1.5 mL/min in one year in both age groups. This is about two times lower than estimate din heavy proteinuric DKD patients (3.9 mL/min-year) and close to the estimated in the general population [45,46]. As the study shows, there was a significant increase in eGFR during the Run-In phase (1.1 mL/min) related to the initiation of LPD and with a lower exogenous creatinine intake. Subsequently, an improvement in eGFR between months 6 and 9 was also noted, probably due to antihypertensive therapy adjustment, with the substitution of RAASi by furosemide, which lead to an increase in intraglomerular pressure. The reduction in proteinuria and in the decline of eGFR were directly associated with a lower protein intake, suggesting that LPD can postpone KRT. The underlying mechanism may be that LPD seems to have a similar hemodynamic effect as RAASi, thus reducing the glomerular hyperfiltration, the main cause of glomerular injury in DKD [47].

Blood pressure control improved in the Intervention phase in both elderly and non-elderly groups. In this sub-analysis, a significant decrease in MAP was noted (−11 mmHg) with a better overall control of blood pressure (median BP 130/60 mmHg) obtained by careful patient monitorization. To prevent further decline in eGFR, RAASi was substituted by diuretics. Dietary sodium restriction also contributed to blood pressure control. As MAP and eGFR did not change following therapeutic regimen alteration, anti-hypertensive therapy adjustments seem appropriate.

Moreover, there was a strong correlation between uncontrolled MAP and incidence of vascular events, similar to previously published data [48]. Vascular events occurred in 15% of the elderly patients, without any significant difference compared to non-elderly patients. In a model of binary regression, the presence of vascular events was correlated with more advanced CKD and uncontrolled MAP. Nutritional intervention did not influence the incidence of vascular events, supporting LPD safety in elderly patients with DKD. However, conflicting results were reported by some papers that found an increased risk of cardiovascular mortality in elderly patients with protein restriction [23].

The safety of the nutritional intervention is always under debate, mainly in the elderly. Our results show that nutritional status improved during the study, with a significant decrease in BMI, without a change in the SGA score; moreover, serum albumin increased and inflammation was lowered, results that are supported by other literature reports [12]. The decrease in BMI was secondary to an improved lifestyle, not as a marker of protein energy wasting. More than that, both glucose metabolism and energy intake remained similar to the recommended target at every study moment, without any difference between elderly and non-elderly patients, suggesting that during this study, patients remained compliant not only to the LPD, but also to the low-carbohydrate diet.

No patients from the elderly group needed KRT, and no patients died.

Thus, LPD seems safe in elderly patients with advanced DKD. However, some studies suggest that even if LPD seems to be nutritionally safe for the elderly, there seems to be a significant correlation between the risk of nutritional status decline, age, and the presence of multiple comorbidities [49,50]. Another study reported that in patients with G4–G5 CKD, the degradation of nutritional status is a more important predictor of renal events than protein intake, highlighting the importance of nutritional status monitoring in CKD patients [51].

These data, as well as the results of the current study, underline the importance of careful patient selection and close monitoring as essential for the safety of a low-protein diet.

## 5. Limitations

This prospective interventional study has some limitations which must be pointed out.

First of all, there was no control group and because of that it is difficult to assess the independent effect of LPD on postponing KRT. Thus, it can be assumed the effect is probably due to a multifactorial approach—controlling T2DM, managing CKD complications, and adopting lifestyle changes including LPD.

Secondly, it included only Caucasian patients with good nutritional status, with well-controlled blood pressure and optimal estimated compliance to diet. Thus, the applicability of the results might be limited.

Additionally, the number of patients is relatively small for the intended effect.

Lastly, the involvement of a single center can be considered a limitation, but this is overshadowed by the years of practical experience in counselling and nutritional monitoring when it comes to the topic in question.

Statistical power is limited by the number of participants, but our cohort had a number of subjects similar to those reported in other studies, reflecting the difficulties in recruiting patients. Although in the future, the prevalence of elderly patients with DKD is expected to increase.

## 6. Conclusions

Dietary protein restriction supplemented with ketoanalogues of the essential amino acids seems to be feasible, efficient, and safe in postponing dialysis in elderly patients with advanced DKD, mainly through better metabolic control while at the same time avoiding malnutrition. However, further randomized studies should be conducted to investigate the independent effect of LPD on lowering kidney function decline or metabolic complications due to CKD in elderly patients.

This approach could significantly slow the decline in renal function for compliant diabetic patients with advanced CKD, regardless of age, without the risk of malnutrition.

## Figures and Tables

**Table 1:** Elderly patients characteristics at Inclusion.

Age (years)	75 (71 to 80)
Sex (male %) *	64
Proteinuria (g/g creatinine)	4.8 (4.6 to 5.2)
eGFR (mL/min)	11.7 (11.2 to 12.2)
Slope of eGFR (mL/min per month) ^i^	−0.49 (−0.59 to −0.39)
Systolic blood pressure	130 (110 to 150)
Diastolic blood pressure	70 (65 to 80)
Mean arterial pressure (mmHg)	93 (83 to 102)
Pulse pressure (mmHg)	70 (50 to 75)
Vascular events (%) *	0
Body mass index (kg/m^2^)	27.1 (25.8 to 28.2)
Subjective global assessment A (%) *	100
Serum albumin (g/dL)	3.9 (3.8 to 4.1)
C-reactive protein (mg/L)	13 (12 to 15)
Glycated hemoglobin (%) *	8.4 (8.0 to 8.7)
Estimated protein intake (g/kg-day)	0.90 (0.84 to 0.97)
RAASi (%) *	90
Furosemide (%) *	51

Data are presented as median and 95% confidence interval (95% CI). * Categorical data are presented as percentages. ^i^ Slope was calculated during the Evaluation phase. eGFR—estimated glomerular filtration rate; RAASI—renin–angiotensin–aldosterone system inhibitors.

**Table 2:** Study parameters by age group at study moments.

	Baseline			End of Study		
	Non-Elderly Patients (n = 53)	Elderly Patients (n = 39)	*p*	Non-Elderly Patients (n = 53)	Elderly Patients (n = 39)	*p*
**Demographic characteristics**						
Age (years)	57 (53 to 58)	75 (72 to 80)	<0.0001			
Sex (male, %) *	68	64	0.70			
**Efficacy parameters**						
Proteinuria (g/g creatinine)	5.2 (5.0 to 5.3)	5.1 (4.8 to 5.3)	0.65	1.6 (1.3 to 1.8)	1.5 (1.0 to 1.8)	0.44
Slope of proteinuria (g/g per month)	−0.05 (−0.37 to 0.17) ^i^	0.00 (−0.40 to 0.17) ^i^	0.52	−0.29 (−0.32 to −0.26) ^ii^	−0.32 (−0.38 to −0.27) ^ii^	0.20
eGFR (mL/min)	12.6 (11.6 to 13.6)	12.6 (11.2 to 13.8)	0.38	11.0 (10.4 to 12.0)	10.7 (9.8 to 11.7)	0.20
Slope of eGFR (mL/min per month)	0.20 (0.14 to 0.49) ^i^	0.22 (0.16 to 0.41) ^i^	0.98	−0.12 (−0.25 to −0.08) ^ii^	−0.10 (−0.15 to −0.08) ^ii^	0.24
**Secondary parameters**						
S-BP (mmHg)	135 (105 to 140)	140 (120 to 160)	0.10	110 (105 to 130)	130 (115 to 145)	0.11
D-BP (mmHg)	85 (75 to 90)	75 (65 to 80)	0.008	65 (65 to 70)	60 (60 to 65)	0.04
MAP (mmHg)	99 (90 to 109)	99 (90 to 109)	0.80	88 (85 to 91)	86 (79 to 91)	0.96
Vascular events (%) *	0	0	-	22.6	15.4	0.39
Cardiovascular events (%)*	0	0	-	13.2	2.6	0.07
Cerebrovascular events (%) *	0	0	-	13.2	12.8	0.96
**Safety parameters**						
BMI (kg/m^2^)	27.3 (25.9 to 29.0)	27.1 (25.5 to 28.1)	0.58	26.5 (25.1 to 27.1)	25.7 (24.7 to 27.0)	0.49
SGA (A, %) *	100	100	-	100	100	-
Serum albumin (g/dL)	4.0 (3.8 to 4.0)	3.9 (3.9 to 4.0)	0.71	4.1 (4.1 to 4.2)	4.2 (4.0 to 4.3)	0.14
CRP (mg/L)	14 (12 to 14)	14 (12 to 15)	0.49	9 (8 to 10)	8 (7 to 9)	0.31
Glycated hemoglobin (%) *	8.1 (8.0 to 8.4)	8.1 (8.0 to 8.3)	0.32	8.1 (7.8 to 8.4)	8.1 (7.8 to 8.3)	0.95
Estimated energy intake (kcal/kg-day)	31.3 (30.2 to 32.6)	31.2 (30.2 to 32.6)	0.69	30.0 (28.5 to 31.8)	31.3 (28.5 to 33.0)	0.46
**Adherence to diet**						
Estimated protein intake (g/kg-day)	0.68 (0.65 to 0.70)	0.68 (0.65 to 0.69)	0.71	0.64 (0.63 to 0.66)	0.64 (0.63 to 0.67)	0.91
**Therapy**						
RAASI (%) *	100	100	-	79.2	69.2	0.27
Furosemide (%) *	64.2	59	0.61	84.9	89.7	0.50

Data are presented as median and 95% confidence interval (95% CI). * Categorical data are presented as percentages. ^i^ Slopes were calculated during the Run-In phase. ^ii^ Slopes were calculated during the Intervention phase. BMI—body mass index; CRP—C-Reactive Protein; D-BP—diastolic blood pressure; eGFR—estimated glomerular filtration rate; MAP—mean arterial blood pressure; RAASI—renin–angiotensin–aldosterone system inhibitors; S-BP—systolic blood pressure; SGA—Subjective Global Assessment.

**Table 3:** Variations in study parameters by age group.

	End of Study—Baseline Difference (Elderly Patients)	Sig.	End of Study—Baseline Difference (Non-Elderly Patients)	Sig.	Sig. ^Δ^
**Efficacy parameters**					
Proteinuria (g/g creatinine)	−3.6 (−3.8 to −3.1)	<0.0001	−3.4 (−3.8 to −3.2)	<0.0001	0.91
eGFR (mL/min)	−1.5 (−1.9 to −1.1)	<0.0001	−1.5 (−1.9 to −1.1)	<0.0001	0.94
**Secondary parameters**					
Systolic blood pressure (mmHg)	−10 (−40 to 8)	0.10	−10 (−25 to 10)	0.26	0.66
Diastolic blood pressure (mmHg)	−10 (−15 to −5)	<0.0001	−15 (−15 to −10)	<0.0001	0.17
Mean arterial pressure (mmHg)	−11 (−19 to −7)	<0.0001	−11 (−24 to −6)	0.002	0.83
Vascular events (%) *	15.4	0.01	23	0.0005	0.39
Cardiovascular events (%) *	2.6	0.32	13	0.008	0.07
Cerebrovascular events (%) *	12.8	0.03	13	0.008	0.96
**Safety parameters**					
Body mass index (kg/m^2^)	−1.0 (−1.9 to −0.6)	0.0003	−1.2 (−1.6 to −0.4)	<0.0001	0.96
Subjective global assessment A (%)	0	-	0	-	-
Serum albumin (g/dL)	0.22 (0.0 to 0.35)	0.02	0.2 (0.1 to 0.4)	0.002	0.66
C-reactive protein (mg/L)	−5 (−7 to −3)	<0.0001	−4 (−6 to −3)	<0.0001	0.26
Glycated hemoglobin (%)	−0.03 (−0.6 to 0.2)	0.87	−0.3 (−0.9 to 0.2)	0.09	0.21
Estimated energy intake (kcal/kg-day)	−0.3 (−2.7 to 2.7)	1	−0.3 (−3.0 to 0.8)	1	0.61
Adherence to energy intake (%) *	2	0.76	2	0.78	0.92
**Adherence the diet**					
Estimated protein intake (g/kg-day)	−0.03 (−0.05 to 0.00)	0.02	−0.05 (−0.06 to 0.01)	0.09	0.73
Adherence to protein restriction (%) *	38.5	0.001	28.3	0.01	0.36
**Therapy**					
RAASi (% patients) *	−31	0.0005	−21	0.0009	0.27
Furosemide (% patients) *	31	0.003	21	0.02	0.84

Data are presented as median and 95% confidence interval (95% CI). * Categorical data are presented as percentages. ^Δ^ Differences between the variations in study parameters by age group. eGFR—estimated glomerular filtration rate; RAASi—renin–angiotensin–aldosterone inhibitors.

**Table 4:** Interaction of efficacy parameters by study moments and group age.

		Proteinuria			eGFR		
	*df*	SS	F	*p*	SS	F	*p*
Elderly	1	0.04	0.58	0.45	0.08	1.92	<0.0001
Study moment	1	6.51	106.57	<0.0001	0.22	5.39	0.17
Elderly × Study moment	1	0.0001	0.002	0.97	0.001	0.02	0.02
Model	3	6.73	36.74	<0.001	0.35	2.88	0.04
Error	548	21.92			33.46		

Two-way ANOVA model. Dependent variables: eGFR and Proteinuria—log transformed. Fixed factors: Elderly (Yes/No), Study Moment (Run-In/Intervention).

**Table 5:** Factors associated with proteinuria in elderly and in non-elderly.

Elderly	B	SE	Beta	95% CI		Sig.
(Constant)	−1.81	0.83		−3.46	−0.16	0.03
eGFR	0.86	0.09	0.60	0.69	1.03	<0.0001
Estimated protein intake	0.75	0.20	0.23	0.35	1.14	<0.0001
Glycated hemoglobin	0.64	0.31	0.13	0.04	1.25	0.04
Body mass index	0.54	0.45	0.07	−0.35	1.44	0.23
Mean arterial blood pressure	0.11	0.20	0.03	−0.29	0.50	0.60
Model of linear regression; adjusted R^2^ = 0.48; *p* ≤ 0.0001 Selecting cases for Elderly						
Non-elderly	B	SE	Beta	95% CI		Sig.
(Constant)	−2.86	0.67		−4.17	−1.55	<0.0001
eGFR	0.71	0.07	0.56	0.57	0.84	<0.0001
Estimated protein intake	0.57	0.17	0.18	0.24	0.89	0.001
Body mass index	1.28	0.38	0.18	0.54	2.02	0.001
Mean arterial blood pressure	0.40	0.21	0.10	−0.02	0.81	0.06
Glycated hemoglobin	0.14	0.26	0.03	−0.38	0.66	0.60
Model of linear regression; adjusted R^2^ = 0.49; *p* ≤ 0.0001 Selecting cases for Non-elderly						

Dependent variable: Proteinuria. Only independent variables significantly correlated with proteinuria were selected. All variables were log transformed to optimize accuracy.

**Table 6:** Factors associated to glomerular filtration rate in elderly and in non-elderly patients.

Elderly	B	SE	Beta	95% CI		Sig.
(Constant)	0.49	0.29		−0.09	1.06	0.10
Proteinuria	0.47	0.05	0.67	0.38	0.56	<0.0001
Estimated protein intake	−0.34	0.15	−0.15	−0.64	−0.05	0.02
Mean arterial blood pressure	0.11	0.15	0.05	−0.18	0.41	0.45
Model of linear regression adjusted R^2^ = 0.41; *p* ≤ 0.0001; Selecting cases for Elderly						
Non-elderly	B	SE	Beta	95% CI		Sig.
(Constant)	0.27	0.35		−0.41	0.95	0.44
Proteinuria	0.47	0.05	0.60	0.38	0.57	<0.0001
Estimated protein intake	−0.22	0.14	−0.09	−0.49	0.06	0.12
Mean arterial blood pressure	0.24	0.18	0.08	−0.11	0.59	0.18
Model of linear regression adjusted R^2^ = 0.36; *p* = 0.000; Selecting cases for Non-elderly						

Dependent variable: eGFR. Only independent variables significantly corelated with proteinuria were selected. All variables were log transformed to optimize accuracy.

**Table 7:** Determinants of vascular events.

	B ± S.E.	Exp(B) (95% CI)	Sig.
eGFR	−5.28 ± 1.48	0.01 (0.00 to 0.09)	0.00
Mean arterial pressure	1.06 ± 0.51	2.88 (1.07 to 7.74)	0.04
Body mass index	0.33 ± 0.38	1.38 (0.66 to 2.90)	0.39
Glycated hemoglobin	0.67 ± 0.43	1.96 (0.85 to 4.51)	0.11
Estimated protein intake	−0.27 ± 1.06	0.77 (0.10 to 5.96)	0.80
Estimated energy intake	−0.54 ± 0.36	0.59 (0.29 to 1.18)	0.13
Elderly *	0.81 ± 0.65	2.24 (0.63 to 7.94)	0.21
Constant	−9.35 ± 2.22	0.00	0.00

Binary logistic regression: Vascular events (Yes/No). * Categorical variable: Elderly (Yes/No). All continuous variables were Z score standardized. Nagelkerke R Square 0.46, *p* < 0.0001; Hosmer and Lemeshow Test *p* = 0.99; The model correctly predicts the difference in 91% of cases.

## Data Availability

The original contributions presented in the study are included in the article. Further inquiries can be directed to the corresponding author.
